# Perception of Plagiarism Among Medical Postgraduate Students: An Observational Study

**DOI:** 10.7759/cureus.64513

**Published:** 2024-07-14

**Authors:** Vijaya L Patil, Praveen Ganganahalli

**Affiliations:** 1 General Surgery, Shri. B. M. Patil Medical College, Hospital, and Research Centre, BLDE (Deemed to be University), Vijayapura, IND; 2 Community Medicine, Shri. B. M. Patil Medical College, Hospital, and Research Centre, BLDE (Deemed to be University), Vijayapura, IND

**Keywords:** article, education, perception, post-graduate students, plagiarism

## Abstract

Introduction

Plagiarism is appropriating another person’s ideas, words, results, or processes without giving appropriate credit and usually claiming them to be one's own. Thus, plagiarism is a dishonest act of fraud or cheating.

Objectives

The objective of this study is to assess the perception of plagiarism among medical postgraduate (PG) students.

Materials & Methods:

An educational observational study was conducted among second-year PG students about the perception of plagiarism by using pre-test and post-test questionnaires after an orientation session on plagiarism and data analysis before the start of dissertation analysis. Questions included were on awareness and attitude towards plagiarism.

Results

A survey involving 91 PG students assessed their understanding of plagiarism. Remarkably, the majority (97.7%) demonstrated awareness of plagiarism, yet only 18.6% had authored a published article. It was discovered that about 30% of the students had resorted to plagiarism at some point during their academic pursuits. Approximately 70.9% of the PG students were acquainted with the University’s plagiarism policy. The survey highlighted a notable enhancement in plagiarism awareness among PG students, with their attitudes toward plagiarism evolving after participating in the session.

Conclusion

Plagiarism can be avoided by implementing rigorous guidelines, ensuring strict policy adherence, and providing comprehensive training before commencing work. Training, retraining, and strict institute policies will help increase awareness about plagiarism and reduce the percentage of plagiarism in scientific writing.

## Introduction

Plagiarism is when someone takes ideas, processes, discoveries, or language from someone else without giving them credit and passes them off as their own work. This dishonest behaviour is a form of academic misconduct or intellectual dishonesty [1]. The importance of professional behaviour and attitude is increasingly recognized in medical education. Instances of unprofessional conduct among physicians can have severe implications for patient safety, disrupt collaborative dynamics, and cause distress. Additionally, graduating medical students who plagiarise may enter the medical profession without the necessary knowledge for competent practice [2-4].

Plagiarism has witnessed a notable rise recently, mainly due to the ubiquitous use of the internet and the easy availability of extensive online information databases. This surge in plagiarism, especially prevalent in postgraduate (PG) master’s programs in medical science, is profoundly concerning and carries extensive ramifications. Its impact is deeply felt, resulting in a substantial devaluation of academic accomplishments and causing considerable distress and disillusionment within the academic circles [1].

The alarming surge in the “publish or perish” culture has led to a notable rise in plagiarism cases. Contributing factors include inadequate language skills, insufficient training in scientific writing, pressure from educational institutions to produce articles, ignorance of the potential repercussions of plagiarism, the ease of accessing online materials, and the temptation to bolster publication numbers hastily without substantial effort [5].

While PG students must undertake a scientific research project and present a dissertation, there needs to be more clarity concerning the level of doctors’ awareness regarding plagiarism. Limited comprehension poses a considerable threat of inadvertent plagiarism among them [6].

The escalating occurrence of plagiarism in institutes and universities worldwide demands immediate attention from faculty members. Neglecting this issue heightens the likelihood of students resorting to plagiarism. Against this backdrop, an assessment survey was devised to investigate the awareness and attitudes regarding plagiarism among PG students at a medical university.

## Materials and methods

An educational observational (cross-sectional) study was conducted among the study population of second-year PG students enrolled in Shri. B.M. Patil Medical College, Vijayapura, Karnataka, India, just before commencing their dissertation data analysis. The study was approved by BLDE (Deemed to be University), Vijayappura, India (approval number: BLDE(DU)/IEC/643/2022-23).

A one-day orientation hands-on training session on data analysis and plagiarism was conducted and facilitated by field experts for second-year PG students. The session included the definition of plagiarism, the acts that constitute plagiarism, the detection and prevention of plagiarism, and the impact of plagiarism with hands-on training in the use of plagiarism detection software.

All second-year PG students who attended the plagiarism session were included and those who were not present for the session or who did not submit responses were excluded from the study. Convenience sampling techniques were used to enrol all the students present for the session. Hence, no sample size was calculated.

Study tool

The questionnaire used was original and validated with the help of health profession education experts, after which it was modified and implemented on the students. Two sets of questionnaires were prepared, one set on knowledge and another on perception of plagiarism.

Study method

A pre-test and post-test questionnaire were administered to the students to assess the session’s effectiveness using a Google Forms link (Google LLC, Mountain View, California, United States). A pre-structured questionnaire proforma on knowledge and perception regarding plagiarism was distributed to the PG students 10 minutes before the session began and again after its completion.

Statistical data analysis

The collected data were compiled into an Excel sheet (Microsoft Corporation, Redmond, Washington, United States) and analysed for frequency distribution (percentage). The significance of differences between the pre-session and post-session responses was evaluated using the chi-square test (p<0.05 was considered significant).

## Results

**Figure 1:**
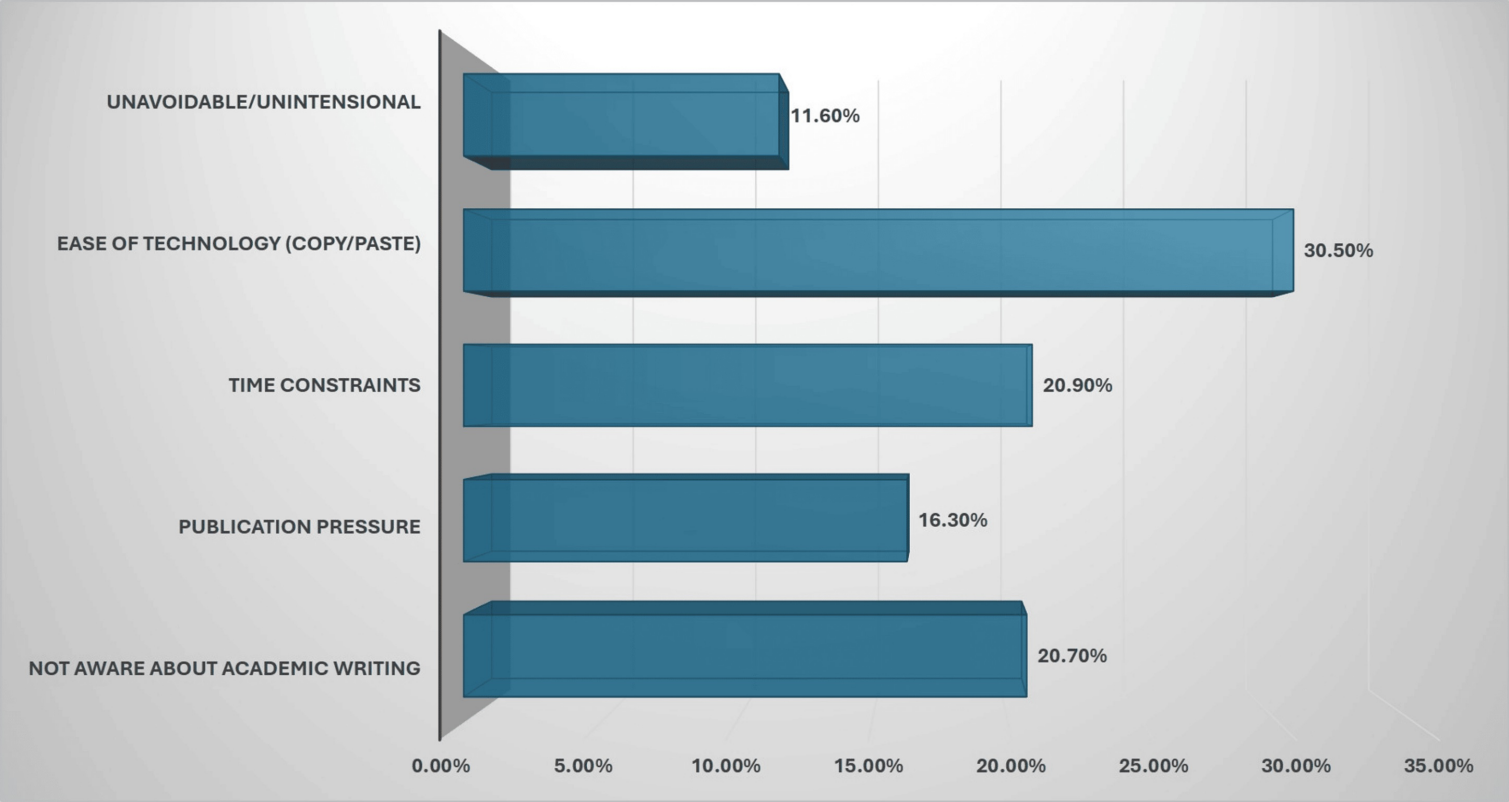
Proportion of most common reasons for plagiarism among postgraduate students (n=91)

A total of 91 PG students participated by attending and submitting the survey response, of which 46 (51%) were female and 45 (49%) were male. Eighty-nine (97.7%) students were aware of plagiarism, but only 17 (18.6%) had published an article. Approximately 27 (30%) students admitted to plagiarising at least once during their academic careers. Surprisingly, around 65 (70.9%) PG students were familiar with the University’s plagiarism policy. The most common reasons for plagiarism were ease of technology by 28 (30.5%) students, time constraints by 19 (20.9%) students, and the compulsion of publication during the course, as depicted in Figure 1.

The survey indicated a notable rise in PG students’ awareness of plagiarism, with their perspectives shifting after participating in the session (Tables 1, 2). This was evident in their altered views on plagiarism-related behaviours, such as submitting papers to multiple journals simultaneously by 14 (15%) students or without co-authors’ consent by 14 (15%) students. Table 1 shows the awareness level regarding plagiarism among PG students before the session increased significantly after the session including instances of plagiarism, plagiarism as a punishable act, the percentage of plagiarism acceptable, and the definition of plagiarism. Table 2 shows the perception level regarding plagiarism among PG students and the change in the students’ attitudes regarding plagiarism in the research and publication process including facts such as plagiarism is an offence or crime, plagiarism cannot be justifiable, and appropriate citations are needed. 

**Table 1:** Awareness regarding plagiarism among the postgraduate students who participated in the study (N=91) *statistically significant (p<0.05)

Variables		Pre-test, % (n)	Post-test, % (n)	Χ^2^ value (p-value)
Any instances when plagiarism could be acceptable	Never acceptable	38.4% (35)	40.7% (37)	1.267 (p=0.73)
	Self-plagiarism with auto-citation	3.5% (3)	05% (5)	
	Using direct quotes with credit to the author	9.3% (8)	4.8% (4)	
	Both b & c	48.8% (44)	49.5% (45)	
Plagiarism is publishable	Yes	77.9% (71)	94.5% (86)	12.37 (p=0.004)*
	No	22.1% (20)	5.5% (5)	
Which of these following practices are considered plagiarism	Paraphrasing without citation	15.1% (14)	12.1% (11)	2.48 (0.47)
	Falsification	1.8% (2)	03% (3)	
	Self-plagiarism	4% (4)	1.4% (1)	
	All the above	79.1% (72)	83.5% (76)	
Which of these following practices is Not considered plagiarism	Purchasing pre-written paper	14% (13)	14.3% (13)	2.95 (0.39)
	Using direct quotes and giving credit	47.7% (43)	53.8% (49)	
	Letting someone else write a paper for your work	11.6% (11)	15.4% (14)	
	Self-plagiarism	26.7% (24)	16.5% (15)	
What percentage of plagiarism is acceptable by most journals?	10%	82.6% (75)	93.4% (85)	10.41 (0.01)*
	20%	12.8% (12)	1.6% (1)	
	25%	3.5% (3)	02% (2)	
	30%	1.1% (1)	03% (3)	
Re-printing one's own previous work may not be considered plagiarism	Agree	47.7% (43)	39.6% (36)	15.22 (0.005)*
	Disagree	32.6% (30)	54.9% (50)	
	Neither agree/disagree	19.8% (18)	5.5% (5)	
Self-plagiarism is not punishable because it is not harmful	Agree	34.9% (32)	24.2% (22)	8.08 (0.01)*
	Disagree	41.9% (38)	61.5% (56)	
	Neither agree/disagree	23.3% (21)	14.3% (13)	
If the work is of good scientific value, the plagiarized parts of a paper may be ignored	Agree	29.1% (26)	22% (20)	4.59 (0.20)
	Disagree	55.8% (51)	69.2% (63)	
	Neither agree/disagree	15.1% (14)	8.8% (8)	

**Table 2:** Perception and attitude regarding plagiarism among the postgraduate students who participated in the study (N=91) *statistically significant (p<0.05)

Variables		Pre-test, % (n)	Post-test, % (n)	Χ^2^ value (p-value)
Plagiarism	Is a crime	27.9% (25)	59.3% (54)	15.86 (0.002)*
	Not normally correct	62.8% (57)	42.9% (39)	
	Does not harm anyone, then it's ok	9.3% (8)	6.6% (6)	
	It is unavoidable	10.5% (10)	6.6% (6)	
It is justified to use one’s own work published without citation to complete the running project	Agree	38.4% (35)	29.7% (27)	2.40 (0.30)
	Disagree	50% (46)	61.5% (56)	
	Neither agree/disagree	11.6% (11)	8.8% (8)	
It is justifiable to copy the author’s permission	Agree	43% (39)	38.5% (35)	2.24 (0.32)
	Disagree	43% (39)	52.7% (48)	
	Neither agree/disagree	14% (13)	8.8% (8)	
The name of the author who plagiarized should be disclosed to scientific committees	Agree	66.3% (60)	63.7% (58)	3.33 (0.18)
	Disagree	17.4% (16)	26.4% (24)	
	Neither agree/disagree	16.3% (15)	9.9% (9)	
Plagiarism should not be considered a serious offence as it is only stealing of words and not tangible assets	Agree	27.9% (25)	24.2% (22)	2.56 (0.27)
	Disagree	53.5% (49)	63.7% (58)	
	Neither agree/disagree	18.6% (17)	12.1% (11)	
It is justifiable to use previous concepts/theories because they remain the same	Agree	52.3% (48)	31.9% (29)	9.47 (0.02)
	Disagree	34.9% (32)	54.9% (50)	
	Neither agree/disagree	12.8% (12)	13.2% (12)	
	No	80.2% (73)	89% (81)	

## Discussion

Engkizar et al. delineated the following eight factors that contribute to plagiarism: inadequate comprehension of plagiarism, the impact of students’ immediate culture on academic task completion, overwhelming academic workload assigned by instructors, students’ lack of enthusiasm for reading, inadequate time for referencing relevant literature, the availability of information technology, restricted purchasing ability, and a lack of proficiency in scientific paper writing [6]. Indian students perceive the University Grant Commission’s stringent guidelines as a good initiative. However, these guidelines cannot be implemented fruitfully without addressing the underlying causes of plagiarism [7].

Kumari et al. examined 160 participants for their study, comprising 93 faculty members and 67 senior residents [8]. The researchers detected a significant difference in the adverse aspect of attitude toward plagiarism across various factors, including departmental affiliation (clinical/para or non-clinical), completion of formal training in medical writing and research ethics, and the underlying motivations for research participation (P < 0.05). This study highlights the critical need to integrate medical writing and research ethics education into undergraduate and postgraduate curricula.

In their study, Javaeed and colleagues discovered that out of 1100 participants, 86.91% (n=956) demonstrated no comprehension of plagiarism [9]. Surprisingly, despite this lack of understanding, a significant majority, 71.18% (n=783), confessed to plagiarism themselves. The predominant form of plagiarism observed among medical students was the replication of content from peers or seniors, facilitated by the ease of sharing and reproducing work within their academic circles. The study underscored a need for more institutional awareness concerning the prevalence of plagiarism, inadequate measures for detection, and a shortage of clear policies addressing the issue, all identified as significant contributing factors.

Bašić et al.'s study revealed that students lacked familiarity with referencing and encountered difficulties with both theoretical and practical aspects [10]. Despite this, they demonstrated positive attitudes towards avoiding plagiarism and maintaining academic integrity, especially in correctly utilizing research publications. 

Kumar et al. made a noteworthy observation regarding recognizing plagiarism issues within academia [11]. They found that “cutting, copying, and pasting text” was the most commonly acknowledged form of plagiarism, scoring an average awareness rating of 4.2±1.28. On the other hand, “collusion or assisting others in plagiarism” received the lowest awareness score of 3.42±1.33. Overall, the findings indicate a troubling lack of attention to plagiarism among respondents. To address this issue, the study proposed several essential measures to aid research scholars in tackling plagiarism, such as ensuring comprehensive referencing, conducting pre-submission plagiarism checks, and fostering dialogues with supervisors and peers.

In their investigation, Parmar et al. delved into the perceptions of plagiarism among faculty members, and PG students, revealing widespread misunderstandings [1]. The findings indicated that 65% of faculty members and 70% of doctoral students perceive direct word-for-word replication as plagiarism. Nonetheless, a considerable portion (35% of faculty and 38% of PG students) mistakenly believe that substituting words is adequate to evade plagiarism. Factors related to the teacher, factors related to the training facility, and factors related to the students were underlined by Bandadi et al., and in their study, 61.3% of participants reported having trouble managing their time while 57% reported not correctly citing sources [12]. Other issues included easy access to information (46.8%), lack of knowledge (51.6%), inadequate training (52%), and a lack of disciplinary actions (76%).

Kallala et al. studied the understanding of plagiarism among dental PG students and found the mean±SD score of the 106 students who participated in the study to be 4.7±2.2, indicating a low level of understanding of plagiarism [13]. In a study by Clarke O et al., out of the 330 students from 40 universities who answered the survey, 75.8% had a good knowledge level (score ≥ 80%) but only 11.6% had a high score in identifying plagiaristic writing (score ≥ 80%) [14]. Most respondents were against plagiarism, with about half saying that it can occasionally be avoided and that self-plagiarism shouldn’t be treated the same as plagiarism of other people’s work. Kampa et al. concluded that the findings of their study helped to create an effective intervention and a robust anti-plagiarism policy for academic institutions, administrators, and policymakers in detecting academic dishonesty while emphasizing the value of integrity in academic pursuit [15].

Students are aware of the adverse effects of plagiarism, not just in terms of grades but also in terms of moral and career consequences. There is a research gap on how digital tools and the internet affect students’ knowledge and practice of plagiarism. This present study helps to understand how simple hands-on training sessions on plagiarism will help to increase awareness and also perception regarding its use. 

The study was limited by being conducted in a single institute and, hence, not generalizable to the entire population of PG students. Conducting a multicentre study to control the study’s limitations is recommended. 

## Conclusions

Avoiding plagiarism necessitates the implementation of stringent guidelines, strict policy adherence, and thorough training. Training, retraining, and strict institute policies will help increase awareness about plagiarism and reduce the percentage of plagiarism in scientific writing. By proactively taking steps to deter plagiarism, we can maintain research integrity and foster the creation of high-calibre work suitable for publication.

## Appendices

Questionnaire 1

Awareness Regarding Plagiarism among Postgraduate Students

1. Any instances when plagiarism could be acceptable: Never Acceptable/Self-plagiarism with auto-citation/Use direct quotes with credit to the author/Both b & c

2. Plagiarism is publishable: Yes/No

3. Which of these following practices is considered plagiarism: Paraphrasing without citation/Falsification/Self-plagiarism/All of the above

4. Which of these following practices is Not considered plagiarism:  Purchasing prewritten paper/Using direct quotes & giving credit/Letting someone else write a paper for your work/Self-plagiarism

5. What percentage of plagiarism is acceptable in most journals?:  10%/20%/25%/30%

6. Re-printing one’s previous work may not be considered plagiarism: Agree/Disagree/Neither agree or disagree

7. Self-Plagiarism is not punishable because it is not harmful:  Agree/Disagree/Neither agree or disagree

8. If the work is of good scientific value, plagiarized parts of the paper may be ignored:  Agree/Disagree/Neither agree or disagree

Perception and Attitude Regarding Plagiarism Among Postgraduate Students

1. Plagiarism:  Is a crime/Not normally correct/If it does not harm anyone, then it’s ok/It is unavoidable

2. It is justified to use one’s work published without citation to complete the running project:  Agree/Disagree/Neither agree or disagree

3. It is justifiable to copy the author’s permission: Agree/Disagree/Neither agree or disagree

4. The name of the author who plagiarized should be disclosed to the scientific committee: Agree/Disagree/Neither agree or disagree

5. Plagiarism should not be considered a serious offence as it is only stealing of words and not tangible assets: Agree/Disagree/Neither agree or disagree

6. It is justifiable to use previous concepts/theories because they remain the same: Agree/Disagree/Neither agree or disagree
